# Potential Genetic Overlap Between Insomnia and Sleep Symptoms in Major Depressive Disorder: A Polygenic Risk Score Analysis

**DOI:** 10.3389/fpsyt.2021.734077

**Published:** 2021-12-03

**Authors:** Lindsay M. Melhuish Beaupre, Arun K. Tiwari, Vanessa F. Gonçalves, Clement C. Zai, Victoria S. Marshe, Cathryn M. Lewis, Nicholas G. Martin, Andrew M. McIntosh, Mark J. Adams, Bernhard T. Baune, Doug F. Levinson, Dorret I. Boomsma, Brenda W. J. H. Penninx, Gerome Breen, Steve Hamilton, Swapnil Awasthi, Stephan Ripke, Lisa Jones, Ian Jones, Enda M. Byrne, Ian B. Hickie, James P. Potash, Jianxin Shi, Myrna M. Weissman, Yuri Milaneschi, Stanley I. Shyn, Eco J. C. de Geus, Gonneke Willemsen, Gregory M. Brown, James L. Kennedy, Major Depressive Disorder Working Group of the Psychiatric Genomics Consortium

**Affiliations:** ^1^Molecular Brain Science Research Department, Centre for Addiction and Mental Health, Campbell Family Mental Health Research Institute, Toronto, ON, Canada; ^2^Institute of Medical Sciences, University of Toronto, Toronto, ON, Canada; ^3^Department of Psychiatry, University of Toronto, Toronto, ON, Canada; ^4^Laboratory Medicine and Pathobiology, University of Toronto, Toronto, ON, Canada; ^5^Social, Genetic and Developmental Psychiatry Centre, King's College London, London, United Kingdom; ^6^Department of Medical and Molecular Genetics, King's College London, London, United Kingdom; ^7^Genetics and Computational Biology, QIMR Berghofer Medical Research Institute, Brisbane, QLD, Australia; ^8^Division of Psychiatry, University of Edinburgh, Edinburgh, United Kingdom; ^9^Department of Psychiatry, University of Münster, Münster, Germany; ^10^Department of Psychiatry, Melbourne Medical School, University of Melbourne, Melbourne, VIC, Australia; ^11^Florey Institute of Neuroscience and Mental Health, University of Melbourne, Melbourne, VIC, Australia; ^12^Department of Psychiatry and Behavioral Sciences, Stanford University, Stanford, CA, United States; ^13^Department of Biological Psychology, Amsterdam Public Health Research Institute, Vrije Universiteit, Amsterdam, Netherlands; ^14^Department of Psychiatry, Amsterdam Public Health and Amsterdam Neuroscience, Amsterdam UMC/Vrije Universiteit, Amsterdam, Netherlands; ^15^National Institute for Health Research (NIHR) Maudsley Biomedical Research Centre, King's College London, London, United Kingdom; ^16^The Permanente Medical Group, San Francisco, CA, United States; ^17^Department of Psychiatry and Psychotherapy, Universitäts Medizin Berlin Campus Charité Mitte, Berlin, Germany; ^18^Analytic and Translational Genetic Unit, Massachusetts General Hospital, Boston, MA, United States; ^19^Medical and Population Genetics, Broad Institute, Cambridge, MA, United States; ^20^Department of Psychiatry, Charité, Berlin, Germany; ^21^Psychological Medicine, University of Worcester, Worcester, United Kingdom; ^22^Medical Research Council (MRC) Centre for Neuropsychiatric Genetics and Genomics, Neuroscience and Mental Health Research Institute, Cardiff University, Cardiff, United Kingdom; ^23^Institute for Molecular Bioscience, University of Queensland, Brisbane, QLD, Australia; ^24^Brain and Mind Centre, University of Sydney, Sydney, NSW, Australia; ^25^Psychiatry Department, University of Iowa, Iowa City, IA, United States; ^26^Division of Cancer Epidemiology and Genetics, National Cancer Institute, Bethesda, MD, United States; ^27^Psychiatry Department, Columbia University College of Physicians and Surgeons, New York, NY, United States; ^28^Division of Epidemiology, New York State Psychiatric Institute, New York, NY, United States; ^29^Washington Permanente Medical Group, Kaiser Permanente Washington Health Research Institute, Seattle, WA, United States

**Keywords:** sleep, major depressive disorder, insomnia, hypersomnia, polygenic risk

## Abstract

**Background:** The prevalence of insomnia and hypersomnia in depressed individuals is substantially higher than that found in the general population. Unfortunately, these concurrent sleep problems can have profound effects on the disease course. Although the full biology of sleep remains to be elucidated, a recent genome-wide association (GWAS) of insomnia, and other sleep traits in over 1 million individuals was recently published and provides many promising hits for genetics of insomnia in a population-based sample.

**Methods:** Using data from the largest available GWAS of insomnia and other sleep traits, we sought to test if sleep variable PRS scores derived from population-based studies predicted sleep variables in samples of depressed cases [Psychiatric Genomics Consortium - Major Depressive Disorder subjects (PGC MDD)]. A leave-one-out analysis was performed to determine the effects that each individual study had on our results.

**Results:** The only significant finding was for insomnia, where *p*-value threshold, *p* = 0.05 was associated with insomnia in our PGC MDD sample (*R*^2^ = 1.75^−3^, *p* = 0.006).

**Conclusion:** Our results reveal that <1% of variance is explained by the variants that cover the two significant *p*-value thresholds, which is in line with the fact that depression and insomnia are both polygenic disorders. To the best of our knowledge, this is the first study to investigate genetic overlap between the general population and a depression sample for insomnia, which has important treatment implications, such as leading to novel drug targets in future research efforts.

## Introduction

Sleep disorders are not an uncommon phenomenon in society. Insomnia, marked by an inadequate amount of sleep or quality of sleep, affects about 17% of Canadians (1). The opposite of insomnia, hypersomnia, is characterized by excessive sleepiness or daytime sleep, affects about 8% of individuals (2, 3). Sleep disturbances are known risk factors for diseases such as coronary heart disease, diabetes and arthritis, and an overall poorer quality of life (4).

A close relationship also exists between sleep disturbances and psychiatric disorders (5). More specifically, research suggests that sleep disturbances can precede the onset of depression (6, 7). Prevalence rates for insomnia and hypersomnia are much higher among depressed individuals population; about 44–88% suffer from insomnia while 25% experience hypersomnia (8). These concurrent sleep problems lead to poorer patient outcomes, including a decreased remission rate, and a greater risk of suicide and suicidal ideation (9–12).

Although the full biology behind sleep remains to be fully elucidated, we do know sleep is, at least partially, regulated by genes via circadian rhythms. Circadian rhythms are believed to be one component of the sleep-wake cycle. They are an internal timing mechanism that uses external stimuli to entrain/maintain a 24-h cycle. This leads to external expression of this internal mechanism. The most prevalent and persistent stimulus for mammals is the light-dark cycle (13, 14). There is a group of genes, known as the core circadian or clock genes, which are part of a series of transcriptional-translational feedback loops that help regulate the circadian rhythmicity (15). Some of the core circadian genes have been implicated in both major depression and accompanying sleep problems, albeit with mixed findings (16).

A recent, genome-wide association study (GWAS) (17) in over 1.3 million individuals from two large studies, the UK Biobank and 23 and Me. In this study, they reported numerous significant loci for insomnia, dozing and napping (characteristic of hypersomnia). Here we use a polygenic risk score (PRS) analysis to determine if hits for these three phenotypes have a shared genetic architecture with insomnia and hypersomnia in individuals diagnosed with major depression.

## Methods

### Study Cohorts

For these analyses we utilized de-identified sleep phenotype and GWAS data on self-identified European-ancestry participants in studies that are part of the Psychiatric Genomics Consortium-Major Depressive Disorder Working Group (PGC MDD) (18), with permission of the investigators. There were 20 studies with appropriate phenotypic data. After excluding studies that we did not have permission to use, that did not have cleaned X-chromosome data, and/or had studies with ID discrepancies between the phenotype and genotype data, we had 13 samples. After implementing quality control procedures (described below), we were left with 11 studies, consisting of *N* = 6,963 MDD cases. These 11 studies were: 1) the Cognitive Function and Mood Study (COFAMS), Genetics of Recurrent Early-Onset Depression Phase II (GENRED2), Netherlands Twin Register/Netherlands Study of Depression and Anxiety (NTR/NESDA), Queensland Institute of Medical Research cohorts (QIMRI610, QIMRI317, and QIMR COEX), RADIANT (German, Irish and US cases) and Sequenced Treatment Alternatives to Relieve Depression (STAR^*^D). All cases were determined to have lifetime MDD diagnosis based on direct interviews or clinician administered DSM-IV checklists (19). All individuals provided informed consent. Ethical clearance was completed for each study across all studies and further details can be found in the individual references for each study (Table 1).

**Table 1:** Sample distribution by study.

**Study name**	**PGC Label**	**Citation**	**Country of origin**	**Sample size (cases)**	**Sleep instrument used**	**Samples included in study**					
						**Insomnia**			**Hypersomnia**		
						**Present (%)**	**Total**	**Female: Male**	**Present (%)**	**Total**	**Female: Male**
COFAMS	COF2	(20)	Australia	120	Hamilton anxiety and depression rating scale (21)	92 (78.63)	117	72:45	8 (6.90)	116	72:44
GenRED2	GRND	(22)	United States of America	830	Diagnostic interview for genetic studies 3.01 MD (22)	465 (63.09)	737	610:127	416 (56.45)	737	610:127
NESDA+NTR	NES1	(23–25)	Netherlands	1494	Composite international diagnostic interview (26)	987 (71.99)	1,371	929:442	569 (41.50)	1,371	929:442
QIMR I610	QI6C	(27)	Australia	499	Composite international diagnostic interview (26)	414 (85.54)	484	336:148	138 (28.63)	482	335:147
QIMR I317	QI3C	(27)	Australia	864	Composite international diagnostic interview (26)	486 (83.94)	579	327:252	83 (14.36)	578	326:252
QIMR COEX	QIO2	(27)	Australia	565	Composite international diagnostic interview (26)	452 (82.48)	548	390:158	108 (19.82)	545	389:156
RADIANT	RAD3	(28)	United Kingdom	1,872	Schedules for clinical assessment in neuropsychiatry (29)	1,144 (71.86)	1,592	1129:463	313 (24.34)	1,286	920:366
RADIANT - German cases	RAGE	(28)	Germany	322	Schedules for clinical assessment in neuropsychiatry (29)	241 (77.00)	313	208:105	31 (10.20)	304	200:104
RADIANT - Irish cases	RAI2	(28)	Ireland	109	Schedules for clinical assessment in neuropsychiatry (29)	14 (66.67)	21	17:4	3 (15.00)	20	15:5
RADIANT - US cases	RAU2	(28)	United States of America	223	Schedules for clinical assessment in neuropsychiatry (29)	18 (62.07)	29	22:7	10 (34.48)	29	19:10
STAR*D	STM2	(30)	United States of America	936	The inventory of depressive symptomology (31, 32)	899 (99.12)	907	543:364	214 (23.59)	907	543:364

COFAMS, Cognitive Function and Mood Study; GENRED2, Genetics of Recurrent Early-Onset Depression Phase II; NESDA, The Netherlands Study of Depression and Anxiety; NTR, Netherlands Twin Registry; QIMR, Queensland Institute for Medical Research; STAR^*^D, Sequenced Treatment Alternatives to Relieve Depression.

### Sleep Phenotype Data

Self-reported sleep data was extracted from two statements from the PGC-MDD phenotype file that were derived from responses to questions in various research interviews or questionnaires about sleep problems during major depressive episodes (Table 1). The first statement was “Insomnia nearly every day,” and the second statement was “Hypersomnia nearly every day.” Outcomes were binary with 1 representing “yes” and 0 representing “no.” Individuals with “NA” for the sleep phenotype data were excluded from the study.

### Genotype Quality Control

We used imputed plink files that were created using the RICOPILI modules provided by PGC-MDD (33). Genotype quality control (QC) procedures were done using PLINK v 1.9 and 2.0 software (34) according to standard genome-wide association procedures.

The following QC procedures were conducted: individuals with sex incongruences were identified (F > 0.8 = male and F < 0.2 = female) and removed (as well as individuals with no self-reported sex data to compare), individual missingness was capped at 2%, all individuals that were related (using identity-by-descent, IBD; pi_hat > 0.2) were removed, and individuals that were +/- 3 standard deviations away from the mean heterozygosity were removed. To determine genetic ancestry, we performed multi-dimensional scaling and a principal components analysis (PCA). We plotted our samples against the 1000 Genomes Phase 3 reference population (35) using R (36) to determine individuals of European ancestry. With our PCA analyses, we removed everyone that was further than +/- 6 standard deviations away from the mean. Finally, individuals with no phenotype data and individuals that overlapped with the UK Biobank data were removed. After these steps we noticed there were *N* = 4 individuals that were missing the sex call in the .fam file, so they were removed.

After merging SNPs that were present in all the samples, SNPs were removed if they did not meet the following parameters: minor allele frequency (MAF; the SNP variant with the lower frequency) 1% or greater, missingness 2% or less, and Hardy-Weinberg equilibrium of 0.000001. In total, 2,484,281 markers were left.

### Insomnia and Hypersomnia (PGC-MDD) Sample Set-Up

We created two datasets based on our phenotype data: (1) Insomnia, coded as MDD4a in the PGC phenotype file (*N* = 6,698), where subjects who had NA for MDD4a (insomnia) were removed and (2) Hypersomnia, coded as MDD4b in the PGC phenotype file (*N* = 6,375), where subjects who had NA for MDD4b (hypersomnia) were removed. Each sample underwent another round of marker QC; SNPs that were not in HWE of 1 × 10^−6^, had MAF of <1% or had missingness >2% were removed. PCA was performed on each sample separately. After plotting the principal components (PCs), a scree plot revealed that the first 3 PCs should be included in our analyses as covariates. Other covariates that were included were sex, and a study indicator.

### Summary Statistics QC

Publicly available summary statistics from the study from the Jansen study (17) on over *N* = 386,000 individuals from the UK Biobank data were used for PRS calculations. More specifically, summary statistics for insomnia, napping and dozing were used. After removing duplicate SNPs, we excluded markers with a MAF <1%. Info scores of all variants included in the summary statistics were already 0.9 or greater.

### Construction of the PRS

Clumping based on linkage disequilibrium performed using the default PRSice settings. A binary case-control PRS was conducted using PRSice using the fast-score option (v2) (37). Next, we extracted the PRS results for each sample across each of the three phenotypes (insomnia, napping, dozing). A linear regression was performed for each independent sample with the PRS scores being the dependent variable (PRS score ~ Sex + 3 PCs) for each of the 8 *p*-value thresholds (*p* = 0.001, 0.05, 0.1, 0.2, 0.3, 0.4, 0.5, 1). A logistic regression was performed on the sample, using the self-reported sleep data as the dependent variable and the standardized residuals as the independent variable. Nagelkerke's *R*^2^ was used to calculate what amount of the variance was explained by the PRS. Bar graphs were plotted using script from the PRSice website (https://choishingwan.github.io/PRS-Tutorial/plink_visual/). The Bonferroni method was applied for multiple testing. Finally, for significant results, a leave-one-out approach was taken to determine if any of the individual studies were driving the results in our total sample.

## Results

The prevalence of insomnia in our sample was 77.8% while hypersomnia was only present in 29.7%.

*N* = 4,583 (68%) of individuals were female in the insomnia sample and *N* = 4,358 (68%) in the hypersomnia sample. Of the females, 77.3% reported insomnia while 22.7% did not; 31.9% of females reported hypersomnia while 68.1% did not. In males, 79% reported insomnia while 21% did not; 25% reported hypersomnia while 75% did not.

### Insomnia

**Figure 1:**
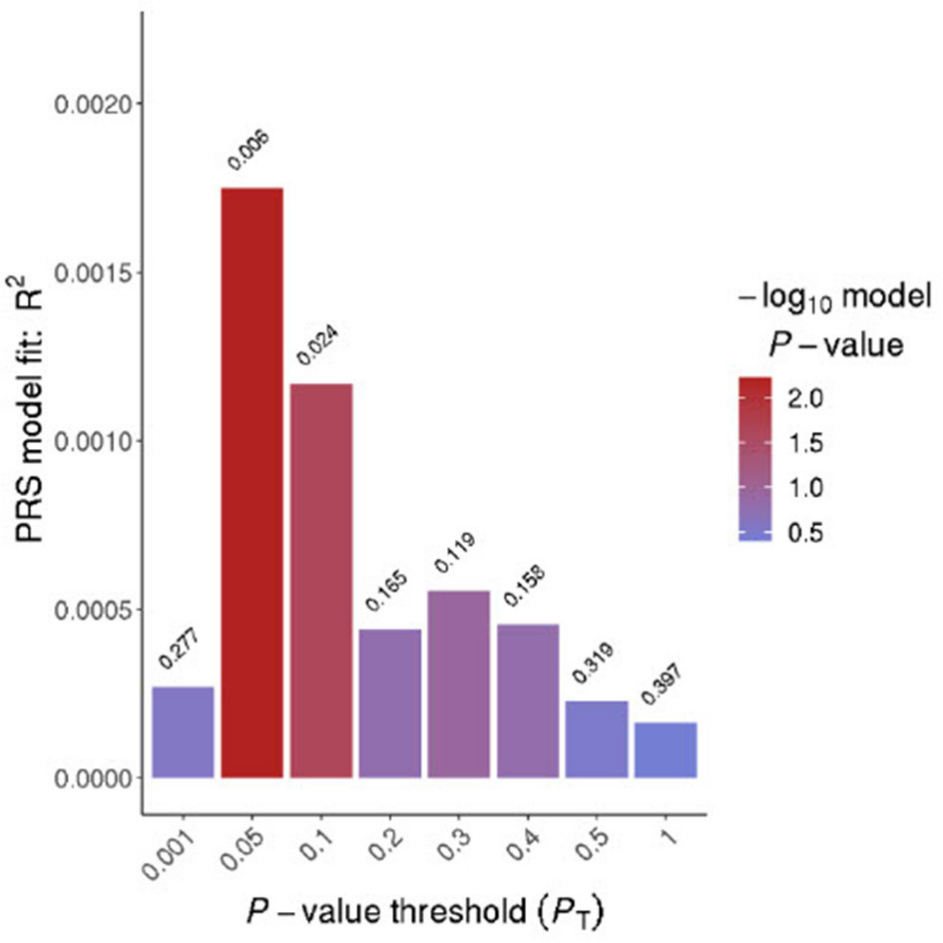
Bar plot for Insomnia. The x-axis displays eight different *p*-value thresholds derived from the fast-score option in PRSice. *R*^2^ represents Nagelkerke's *R*-squared and the values on the top of the bars represent the *p*-values for that threshold.

Prior to applying Bonferroni multiple test corrections (0.05/3), only two of the *p*-value thresholds were significant in our model at a *p* ≤ 0.05, *p* = 0.05 and *p* = 0.1 (Figure 1). The threshold of *p* = 0.05 explained <1% of variance in the risk of insomnia (*R*^2^ = 1.75E-03) (Table 2). The *p* = 0.1 threshold also explained marginal variance (*R*^2^ = 1.17E-03) (Table 2). Only the *p*-value threshold of *p* = 0.05 remained significant after test corrections were applied.

**Table 2:** Insomnia findings.

**Threshold**	***R* ^2^**	***P***	**Coefficient**	**Standard error**	**# of SNPs**
0.001	2.71E-04	0.277	0.032	0.029	844
0.05	1.75E-03	0.006	0.081	0.029	11,008
0.1	1.17E-03	0.024	0.067	0.029	17,846
0.2	4.41E-04	0.165	0.041	0.029	28,837
0.3	5.55E-04	0.119	0.046	0.029	38,202
0.4	4.56E-04	0.158	0.042	0.029	46,391
0.5	2.27E-04	0.319	0.029	0.029	53,454
1	1.64E-04	0.397	0.025	0.029	75,711

*R^2^ represents Nagelkerke's R-squared; P represents the p-value; # represents Number*.

The sensitivity analysis for a *p*-value threshold of *p* = 0.05 revealed that significance increased when QI6C was removed from the analysis (*p* = 0.002) (Table 3). The remainder of results of the leave-one-out analysis can be found in Table 3.

**Table 3:** Leave-one-out analysis results.

	***R^**2**^***	***P***	**Coefficient**	**Standard error**	**Sample size**
COF2	1.43E-03	0.0133	0.07354	0.02971	117
GRND	1.79E-03	0.00915	0.08398	0.03222	737
NES1	2.03E-03	0.00853	0.0891	0.03387	1,371
QI3C	1.94E-03	0.00489	0.08532	0.03032	484
QI6C	2.36E-03	0.00209	0.09411	0.03058	579
QIO2	1.47E-03	0.015	0.07426	0.03054	548
RAD3	1.23E-03	0.0455	0.06969	0.03484	1,592
RAGE	1.70E-03	0.00789	0.08021	0.03019	313
RAI2	1.69E-03	0.00664	0.08017	0.02953	21
RAU2	1.77E-03	0.00558	0.08194	0.02957	29
STM2	1.92E-03	0.00604	0.08295	0.03021	907

*R^2^ represents Nagelkerke's R-squared; P represents the p-value*.

### Hypersomnia

No significant *p*-value thresholds were identified when using summary statistics for “Napping” and “Dozing.” Results for both can be found in Supplementary Tables 1, 2 as well as Supplementary Figures 1, 2.

## Discussion

Simply, polygenic risk scores are the sum of alleles per locus weighted by an estimate of the alleles' effect sizes based on GWAS summary statistics (38, 39). The most popular method, and arguably the most straight-forward is the pruning + thresholding method, which is available using PRSice (v2) software (37). First, SNPs are pruned based on linkage disequilibrium so that one is left with a smaller number of independent SNPs. Second, eight *p*-value cut-off scores are used to construct the eight thresholds (37–39). In order to account for our covariates, and maximize power, we performed a linear regression on the PRS scores for each study, standardized the residuals, and then used the standardized residuals to perform a logistic regression on our phenotypes of interest, insomnia and hypersomnia.

First, we found that the prevalence rates for insomnia were representative of the prevalence rates from other reports (8, 40). The prevalence of hypersomnia in our sample was slightly higher than others have reported (8, 40). Interestingly, the prevalence of insomnia between sexes was similar, but the prevalence of hypersomnia in females was 7% higher than in males. Our results are opposite to prior literature, that suggested depressed males are more likely to experience hypersomnia than depressed females (41). However, their sample was significantly smaller (*N* < 500), which may explain the difference in prevalence rates.

In insomnia, we found that PRS scores for SNPs with a *p*-value threshold of ≤ 0.05 in the insomnia GWAS marginally predicted insomnia in our PGC MDD cohort. However, <1% of variance was explained by these markers. This truly is not surprising given that insomnia is already known to be a polygenic disorder with an estimated SNP-based heritability to be 7% in the UK Biobank sample whose summary statistics we used (17). This is much lower than the heritability of insomnia estimated using twin studies, which suggests 38–59% (17, 42). The substantial range in heritability was due to sex differences, as the heritability of insomnia was much higher in females (59%) than males (38%), which is why we opted to include sex as a covariate (42). Furthermore, there are many sleep-related changes that differ between the sexes, including sex differences in the homeostatic response to sleep as well as slow wave amplitude during non-rapid-eye movement sleep (43, 44).

The other point we would like to note about the insomnia findings comes from our leave-one-out analysis. We noticed that our results became more significant when QI6C was removed from the analysis. Both the *p*-value became more significant (*p* = 0.002) and the variance explained increased (*R*^2^ = 2.36^−03^), which suggests that this dataset is more heterogenous. Indeed, QI6C is a community sample with diagnoses based on self-report questionnaires. Interestingly, QIO2 is also a community sample, recruited in an identical manner to QI6C; but when this sample is removed, significance is decreased. As with all studies, there are a few other limitations to this work. First, the phenotype we are using to characterize insomnia and hypersomnia are based on self-report data. Some studies suggest that self-reported sleep measures differ from objectively measured sleep data and therefore interpretation of data may be more limited (45). Secondly, there are numerous lifestyle factors known to affect sleep for which we were unable to covary. For example, we were unable to include medications in our analyses. Arguably, almost all of the medications on the market today can affect sleep (46). One of the most well-known classes to influence sleep is sleep medications, such as sedative/hypnotics, which are commonly used to treat insomnia (47). Benzodiazepines are another class of medication used to induce sleep (47). Interestingly, in 2010, 20.8 million prescriptions for sleep medication were written in the USA, a 293% increase from the number written in 1999, which confirms how common these sleep medications are used (48). There are also substances that promote insomnia (47). For example, there is extensive evidence that proves that caffeine reduces sleep quality and duration (49). This would likely bias one's subjective opinions of their sleep. Other lifestyle factor that can negatively affect restorative sleep are regular stress and a lack of routine exercise (50). Third, the unique aspect regarding the PGC-MDD sample is that it is comprised of many smaller samples, including variations such as different genotyping platforms. This means, to control for potential sample effects, the sample origin should be included in the analysis, which negatively affects the power of the analyses. A recent study has also suggested seven subtypes of insomnia (51). Although it would have been interesting to investigate these subtypes in our sample, to determine if one has a stronger genetic basis than others, we were unable to do so as we were limited by the phenotype data. In future, we hope to be able to extend upon our results by incorporating these various subtypes into a model. Finally, despite the fact that the sample sizes were similar for both phenotypes of interest (insomnia and hypersomnia), we were only able to provide evidence for genetic overlap with insomnia. We hypothesize our lack of findings for hypersomnia was because we were using GWAS summary statistics for napping and dozing to represent hypersomnia. This does not fully capture hypersomnia, a broad term used for a variety of sleep disorders ranging from narcolepsy to idiopathic hypersomnia.

To the best of our knowledge, this study is the first of its kind to investigate and find genetic overlap between self-reported insomnia in the general population and depressed individuals. This could have important implications on treating MDD and insomnia going forward. Although the results need to be taken with caution as this is a preliminary study and very little variance can be accounted for by the genes implicated in insomnia in the UK Biobank, suggesting that the genetics overlap is minimal. Further research should focus on a similar study design, but using objectively measured sleep data, which should provide more precision for sleep measures. Overall, our results may make helpful contributions toward more accurate diagnosis and personalized treatment.

## Data Availability Statement

The data analyzed in this study is subject to the following licenses/restrictions: The data that support the findings of this study are available from the Psychiatric Genomics Consortium-Major Depressive Disorder (PGC MDD) Working group. Some restrictions apply to the availability of these data. Information on data access is available online at: https://pgcdataaccess.formstack.com/forms/pgc_data_access. Requests to access these datasets should be directed to https://pgcdataaccess.formstack.com/forms/pgc_data_access.

## Ethics Statement

The studies involving human participants were reviewed and approved by the Research Ethics Board at the Centre for Addiction and Mental Health. The patients/participants provided their written informed consent at each site of collection.

## Author Contributions

LM conceptualized the idea, performed the analyses, and prepared the first draft of the manuscript. AT assisted with statistical analysis, results interpretation, and editing of the manuscript. VG helped with the conceptualization of the idea and data analysis. CZ assisted with analysis interpretation and preparation of the manuscript. VM assisted with data analysis. CL helped with data acquisition, statistical analysis, interpretation of results, and manuscript edits. NM helped with data acquisition, statistical analysis, and manuscript edits. AM helped with statistical analyses and manuscript editing. SA and SR helped with data analysis. MA, BB, DL, DB, BP, GB, SH, LJ, IJ, EB, IH, JP, JS, MW, YM, SS, EG, and GW each contributed to data acquisition and provided edits to the manuscript. GMB assisted with results interpretation and manuscript edits. JK helped with the conceptualization of the idea, data analysis, results interpretation, and editing of the manuscript. All authors contributed to the article and approved the submitted version.

## Funding

This project was supported by the Frederick Banting and Charles Best Canada Graduate Scholarship (LM), the Granville Nickerson Fellowship in Pharmacogenetics (AT), Brain and Behavior Research Foundation: NARSAD (AT), McLaughlin Centre Accelerator Grant (2019-2020) (AT), CAMH Foundation (VG), Brain and Behavior Research Foundation (NARSAD Young Investigator) (VG), McLaughlin Centre Accelerator Grant (VG), Larry and Judy Tanenbaum Foundation (JK). The NTR/NESDA dataset was funded by the: Netherlands Organization for Scientific Research (NWO) and MagW/ZonMW grants 904-61-090, 985-10-002, 912-10-020, 904-61-193,480-04-004, 463-06-001, 451-04-034, 400-05-717, Addiction-31160008, Middelgroot-911-09-032, Spinozapremie 56-464-14192, Center for Medical Systems Biology (CSMB, NWO Genomics), Biobanking and Biomolecular Resources Research Infrastructure (BBMRI–NL, 184.021.007); the European Science Foundation (ESF, EU/QLRT-2001-01254), the European Community's Seventh Framework Program (FP7/2007-2013), ENGAGE (HEALTH-F4-2007-201413); the European Science Council (ERC Advanced, 230374), Rutgers University Cell and DNA Repository (NIMH U24 MH068457-06), the Avera Institute, Sioux Falls, South Dakota (USA) and the National Institutes of Health (NIH, R01D0042157-01A, MH081802, Grand Opportunity grants 1RC2 MH089951 and 1RC2 MH089995), the Netherlands Organization for Scientific Research (Geestkracht program grant 10-000-1002); the Center for Medical Systems Biology (CSMB, NWO Genomics), Biobanking and Biomolecular Resources Research Infrastructure (BBMRI-NL), VU University's Institutes for Health and Care Research (EMGO+) and Neuroscience Campus Amsterdam, University Medical Center Groningen, Leiden University Medical Center, National Institutes of Health (NIH, R01D0042157-01A, MH081802, Grand Opportunity grants 1RC2 MH089951 and 1RC2 MH089995), Genetic Association Information Network (GAIN) of the Foundation for the National Institutes of Health. Computing was supported by BiG Grid, the Dutch e-Science Grid, which was financially supported by NWO.

## Conflict of Interest

The authors declare that the research was conducted in the absence of any commercial or financial relationships that could be construed as a potential conflict of interest.

## Publisher's Note

All claims expressed in this article are solely those of the authors and do not necessarily represent those of their affiliated organizations, or those of the publisher, the editors and the reviewers. Any product that may be evaluated in this article, or claim that may be made by its manufacturer, is not guaranteed or endorsed by the publisher.
