# Corrigendum: Potential genetic overlap between insomnia and sleep symptoms in major depressive disorder: A polygenic risk score analysis

**DOI:** 10.3389/fpsyt.2022.893816

**Published:** 2022-08-04

**Authors:** Lindsay M. Melhuish Beaupre, Arun K. Tiwari, Vanessa F. Gonçalves, Clement C. Zai, Victoria S. Marshe, Cathryn M. Lewis, Nicholas G. Martin, Andrew M. McIntosh, Mark J. Adams, Bernhard T. Baune, Doug F. Levinson, Dorret I. Boomsma, Brenda W. J. H. Penninx, Gerome Breen, Steve Hamilton, Swapnil Awasthi, Stephan Ripke, Lisa Jones, Ian Jones, Enda M. Byrne, Ian B. Hickie, James P. Potash, Jianxin Shi, Myrna M. Weissman, Yuri Milaneschi, Stanley I. Shyn, Eco J. C. de Geus, Gonneke Willemsen, Gregory M. Brown, James L. Kennedy, Major Depressive Disorder Working Group of the Psychiatric Genomics Consortium

**Affiliations:** ^1^Molecular Brain Science Research Department, Centre for Addiction and Mental Health, Campbell Family Mental Health Research Institute, Toronto, ON, Canada; ^2^Institute of Medical Sciences, University of Toronto, Toronto, ON, Canada; ^3^Department of Psychiatry, University of Toronto, Toronto, ON, Canada; ^4^Laboratory Medicine and Pathobiology, University of Toronto, Toronto, ON, Canada; ^5^Social, Genetic and Developmental Psychiatry Centre, King's College London, London, United Kingdom; ^6^Department of Medical and Molecular Genetics, King's College London, London, United Kingdom; ^7^Genetics and Computational Biology, QIMR Berghofer Medical Research Institute, Brisbane, QLD, Australia; ^8^Division of Psychiatry, University of Edinburgh, Edinburgh, United Kingdom; ^9^Department of Psychiatry, University of Münster, Münster, Germany; ^10^Department of Psychiatry, Melbourne Medical School, University of Melbourne, Melbourne, VIC, Australia; ^11^Florey Institute of Neuroscience and Mental Health, University of Melbourne, Melbourne, VIC, Australia; ^12^Department of Psychiatry and Behavioral Sciences, Stanford University, Stanford, CA, United States; ^13^Department of Biological Psychology, Amsterdam Public Health Research Institute, Vrije Universiteit, Amsterdam, Netherlands; ^14^Department of Psychiatry, Amsterdam Public Health and Amsterdam Neuroscience, Amsterdam UMC/Vrije Universiteit, Amsterdam, Netherlands; ^15^National Institute for Health Research (NIHR) Maudsley Biomedical Research Centre, King's College London, London, United Kingdom; ^16^The Permanente Medical Group, San Francisco, CA, United States; ^17^Department of Psychiatry and Psychotherapy, Universitäts Medizin Berlin Campus Charité Mitte, Berlin, Germany; ^18^Analytic and Translational Genetic Unit, Massachusetts General Hospital, Boston, MA, United States; ^19^Medical and Population Genetics, Broad Institute, Cambridge, MA, United States; ^20^Department of Psychiatry, Charité, Berlin, Germany; ^21^Psychological Medicine, University of Worcester, Worcester, United Kingdom; ^22^Medical Research Council (MRC) Centre for Neuropsychiatric Genetics and Genomics, Neuroscience and Mental Health Research Institute, Cardiff University, Cardiff, United Kingdom; ^23^Institute for Molecular Bioscience, University of Queensland, Brisbane, QLD, Australia; ^24^Brain and Mind Centre, University of Sydney, Sydney, NSW, Australia; ^25^Psychiatry Department, University of Iowa, Iowa City, IA, United States; ^26^Division of Cancer Epidemiology and Genetics, National Cancer Institute, Bethesda, MD, United States; ^27^Psychiatry Department, Columbia University College of Physicians and Surgeons, New York, NY, United States; ^28^Division of Epidemiology, New York State Psychiatric Institute, New York, NY, United States; ^29^Washington Permanente Medical Group, Kaiser Permanente Washington Health Research Institute, Seattle, WA, United States

**Keywords:** sleep, major depressive disorder, insomnia, hypersomnia, polygenic risk

In the original article, there was an error. The prevalence of hypersomnia in females and males was reversed, such that the prevalence of hypersomnia was reported as the prevalence of no hypersomnia for both sexes.

A correction has been made to **Results**, paragraph one:

“*N* = 4,583 (68%) of individuals were female in the insomnia sample and *N* = 4,358 (68%) in the hypersomnia sample. Of the females, 77.3% of reported insomnia while 22.7% do not; 68.1% of females reported hypersomnia while 31.9% did not. In males, 79% reported insomnia while 21% did not; 75% reported hypersomnia while 25% did not” has been corrected to “*N* = 4,583 (68%) of individuals were female in the insomnia sample and *N* = 4,358 (68%) in the hypersomnia sample. Of the females, 77.3% reported insomnia while 22.7% did not; 31.9% of females reported hypersomnia while 68.1% did not. In males, 79% reported insomnia while 21% did not; 25% reported hypersomnia while 75% did not.”

A correction has been made to **Discussion**, paragraph two:

“First, we found that the prevalence rates for insomnia are representative of the prevalence rates from other reports (8, 40). The prevalence of hypersomnia in our sample is slightly higher than others have reported (8, 40). Interestingly, the prevalence of insomnia between sexes was similar, but the prevalence of hypersomnia in males was 7% higher than in females. The direction of our hypersomnia results is in accordance with prior literature, which suggests depressed males are more likely to experience hypersomnia than depressed females. However, the study also suggests a more significant difference than observed in our results (41). However, their sample was significantly smaller (*N* < 500, which may explain the difference in prevalence rates)” has been corrected to “First, we found that the prevalence rates for insomnia were representative of the prevalence rates from other reports (8, 40). The prevalence of hypersomnia in our sample was slightly higher than others have reported (8, 40). Interestingly, the prevalence of insomnia between sexes was similar, but the prevalence of hypersomnia in females was 7% higher than in males. Our results are opposite to prior literature, that suggested depressed males are more likely to experience hypersomnia than depressed females (41). However, their sample was significantly smaller (*N* < 500), which may explain the difference in prevalence rates.”

In the original article, there were mistakes in the labelling of **Supplementary Tables 1**, **2** as published. Their labelling was reversed, such that **Supplementary Table 1** was referred to as if it were **Supplementary Table 2**, and vice versa. The **Supplementary Material** labelling has been corrected.

In the original article, there was a mistake in the reference order for the citations included in Table 1. Twelve references [i.e., (20–32)] were incorrect. References 20–32 have been corrected and subsequently the full reference list and citations have been updated.

The authors apologize for these errors and state that this does not change the scientific conclusions of the article in any way. The original article has been updated.

## References

[20] Baune BT Air T. Clinical, functional, and biological correlates of cognitive dimensions in major depressive disorder - rationale, design, and characteristics of the cognitive function and mood study (CoFaM-Study). Front Psychiatry. (2016) 7:150. 10.3389/fpsyt.2016.00150 27616997 PMC4999943

[21] Williams JBW. A structured interview guide for the hamilton depression rating scale. Arch Gen Psychiatry. (1988) 45:742–7. 10.1001/archpsyc.1988.01800320058007 3395203

[22] Shi J Potash JB Knowles JA Weissman MM Coryell W Scheftner WA. Genome-wide association study of recurrent early-onset major depressive disorder. Mol Psychiatry. (2011) 16:193–201. 10.1038/mp.2009.124 20125088 PMC6486400

[23] Penninx BWJH Beekman ATF Smit JH Zitman FG Nolen WA Spinhoven P. The Netherlands Study of Depression and Anxiety (NESDA): rationale, objectives and methods. Int J Methods Psychiatr Res. (2008) 17:121–40. 10.1002/mpr 18763692 PMC6878352

[24] Penninx BWJH Eikelenboom M Giltay EJ van Hemert AM Riese H Schoevers RA. Cohort profile of the longitudinal netherlands study of depression and anxiety (NESDA) on etiology, course and consequences of depressive and anxiety disorders. J Affect Disord. (2021) 287:69–77. 10.1016/j.jad.2021.03.026 33773360

[25] Willemsen G Vink JM Abdellaoui A den Braber A van Beek JHDA Draisma HHM. The Adult Netherlands Twin Register: twenty-five years of survey and biological data collection. Twin Res Hum Genet. (2013) 16:271–81. 10.1017/thg.2012.140 23298648 PMC3739974

[26] The World Health Organization. Composite International Diagnostic Interview, Core Version 2.1: Interviewer's Manual. Geneva: World Health Organization (1997).

[27] Wray NR Pergadia ML Blackwood DHR Penninx BWJH Gordon SD Nyholt DR. Genome-wide association study of major depressive disorder: new results, meta-analysis, and lessons learned. Mol Psychiatry. (2012) 17:36–48. 10.1038/mp.2010.109 21042317 PMC3252611

[28] Lewis CM Ng MY Butler AW Cohen-Woods S Uher R Pirlo K. Genome-wide association study of major recurrent depression in the U.K. population. Am J Psychiatry. (2010) 167:949–57. 10.1176/appi.ajp.2010.09091380 20516156

[29] Wing JK Babor T Brugha T Burke J Cooper JE Giel R. SCAN Schedules for clinical assessment in neuropsychiatry. Arch Gen Psychiatry. (1990) 47:589–93. 2190539 10.1001/archpsyc.1990.01810180089012

[30] Shyn SI Shi J Kraft JB Potash JB Knowles JA Weissman MM. Novel loci for major depression identified by genome-wide association study of sequenced treatment alternatives to relieve depression and meta-analysis of three studies. Mol Psychiatry. (2011) 16:202–15. 10.1038/mp.2009.125 20038947 PMC2888856

[31] Trivedi MH Rush AJ Ibrahim HM Carmody TJ Biggs MM Suppes T. The inventory of depressive symptomatology, clinician rating (IDS-C) and self-report (IDS-SR), and the quick inventory depressive symptomatology, clinician rating (QIDS-C) and self-report (QIDS-SR) in public sector patients with mood disorders: A psychome. Psychol Med. (2004) 34:73–82. 10.1017/S0033291703001107 14971628

[32] Sunderajan P Gaynes BN Wisniewski SR Miyahara S Fava M Akingbala F. Insomnia in patients with depression: A STAR^*^D report. CNS Spectr. (2010) 15:394–404. 10.1017/s1092852900029266 20625372

