# Supplementary material for: Potential Genetic Overlap Between Insomnia and Sleep Symptoms in Major Depressive Disorder: A Polygenic Risk Score Analysis
Source: Front Psychiatry. 2021 Dec 3;12:734077. doi: 10.3389/fpsyt.2021.734077 (PMC8678563; doi:10.3389/fpsyt.2021.734077)
Supplement: Supplementary file 1 [file Data_Sheet_1.docx]

Supplementary Material

Supplementary Table 1: PRS Results for Napping

| Threshold | R2 | P | Coefficient | Standard Error | # of SNPs |
| --- | --- | --- | --- | --- | --- |
| 0.001 | 3.59E-05 | 0.688 | -0.01101 | 0.02742 | 370 |
| 0.05 | 0.000123 | 0.457 | -0.0204 | 0.02741 | 9181 |
| 0.1 | 0.000142 | 0.424 | -0.02191 | 0.02741 | 15804 |
| 0.2 | 0.000374 | 0.195 | -0.03554 | 0.02743 | 27042 |
| 0.3 | 0.00024 | 0.3 | -0.02844 | 0.02742 | 36575 |
| 0.4 | 0.000133 | 0.44 | -0.02118 | 0.02741 | 45030 |
| 0.5 | 0.000305 | 0.242 | -0.03207 | 0.02742 | 52488 |
| 1 | 8.25E-05 | 0.543 | -0.01668 | 0.02742 | 75539 |

Supplementary Table 2: PRS Results for Dozing

| Threshold | R2 | P | Coefficient | Standard Error | # of SNPs |
| --- | --- | --- | --- | --- | --- |
| 0.001 | 0.000172 | 0.38 | 0.02407 | 0.02741 | 445 |
| 0.05 | 5.75E-06 | 0.872 | -0.0044 | 0.027406 | 9489 |
| 0.1 | 4.08E-06 | 0.892 | -0.00371 | 0.027411 | 16101 |
| 0.2 | 0.00019 | 0.356 | 0.02531 | 0.02742 | 27348 |
| 0.3 | 1.14E-04 | 0.475 | 0.01959 | 0.02741 | 26893 |
| 0.4 | 8.27E-06 | 0.847 | 0.005279 | 0.027414 | 45214 |
| 0.5 | 3.78E-07 | 0.967 | 0.001129 | 0.027414 | 52507 |
| 1 | 4.32E-06 | 0.889 | -0.00382 | 0.027416 | 75429 |

Supplementary Figure Legends

Supplementary Figure 1: Bar plot for Napping. The eight p-value thresholds derived from the fast-score option in PRSice are displayed along the x-axis. R^2^ represents Nagelkerke’s R-squared and the values on the top of the bars represent the p-values for that threshold

Supplementary Figure 2: Bar plot for Dozing. The eight p-value thresholds along the x-axis are derived from the fast-score option in PRSice. R^2^ represents Nagelkerke’s R-squared and the values on the top of the bars represent the p-values for that threshold
