# Supplementary figures and images for: Potential Genetic Overlap Between Insomnia and Sleep Symptoms in Major Depressive Disorder: A Polygenic Risk Score Analysis
Source: Front Psychiatry. 2021 Dec 3;12:734077. doi: 10.3389/fpsyt.2021.734077 (PMC8678563; doi:10.3389/fpsyt.2021.734077)

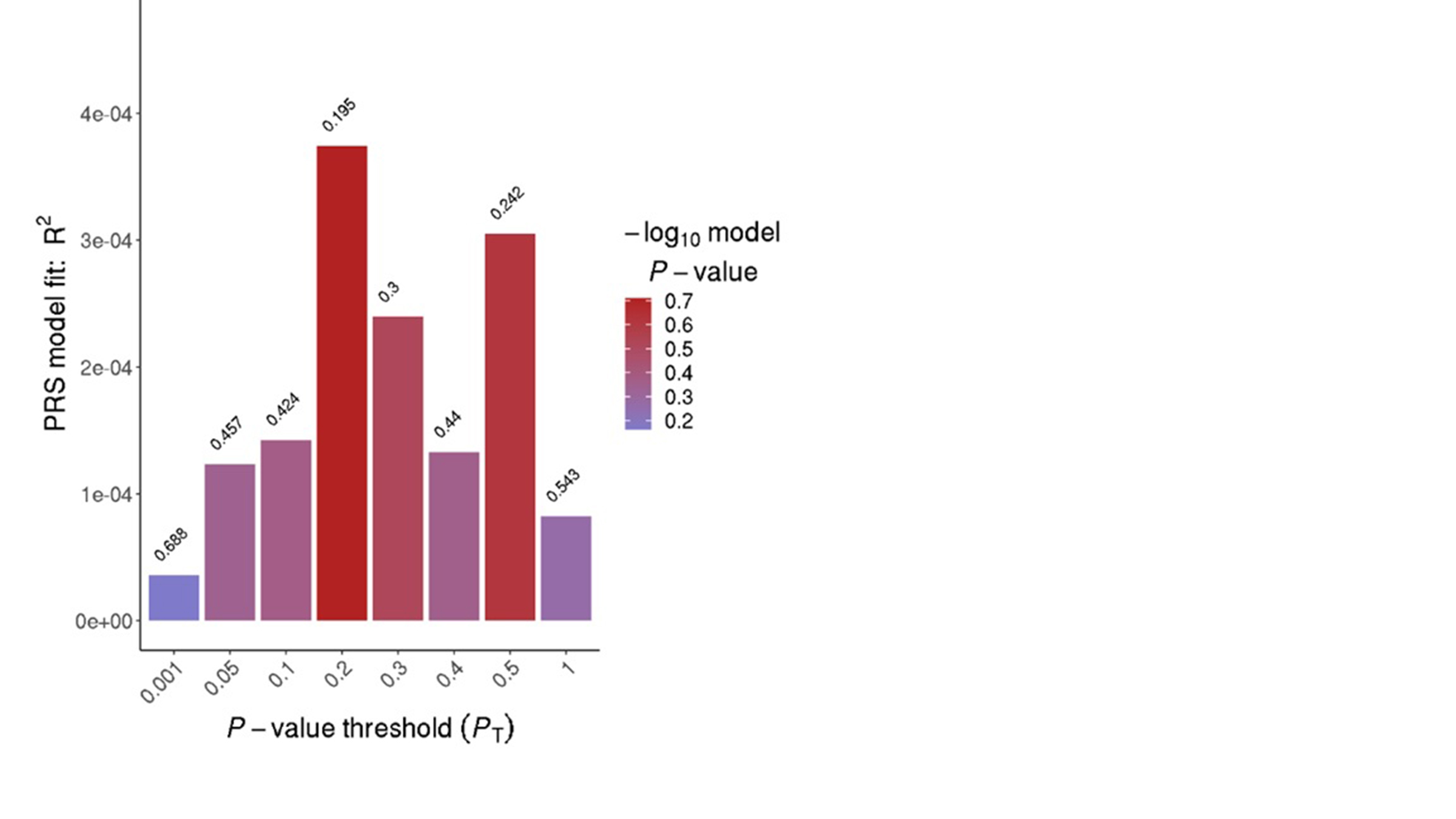

Supplement: Supplementary file 2 [file Image_1.JPEG]

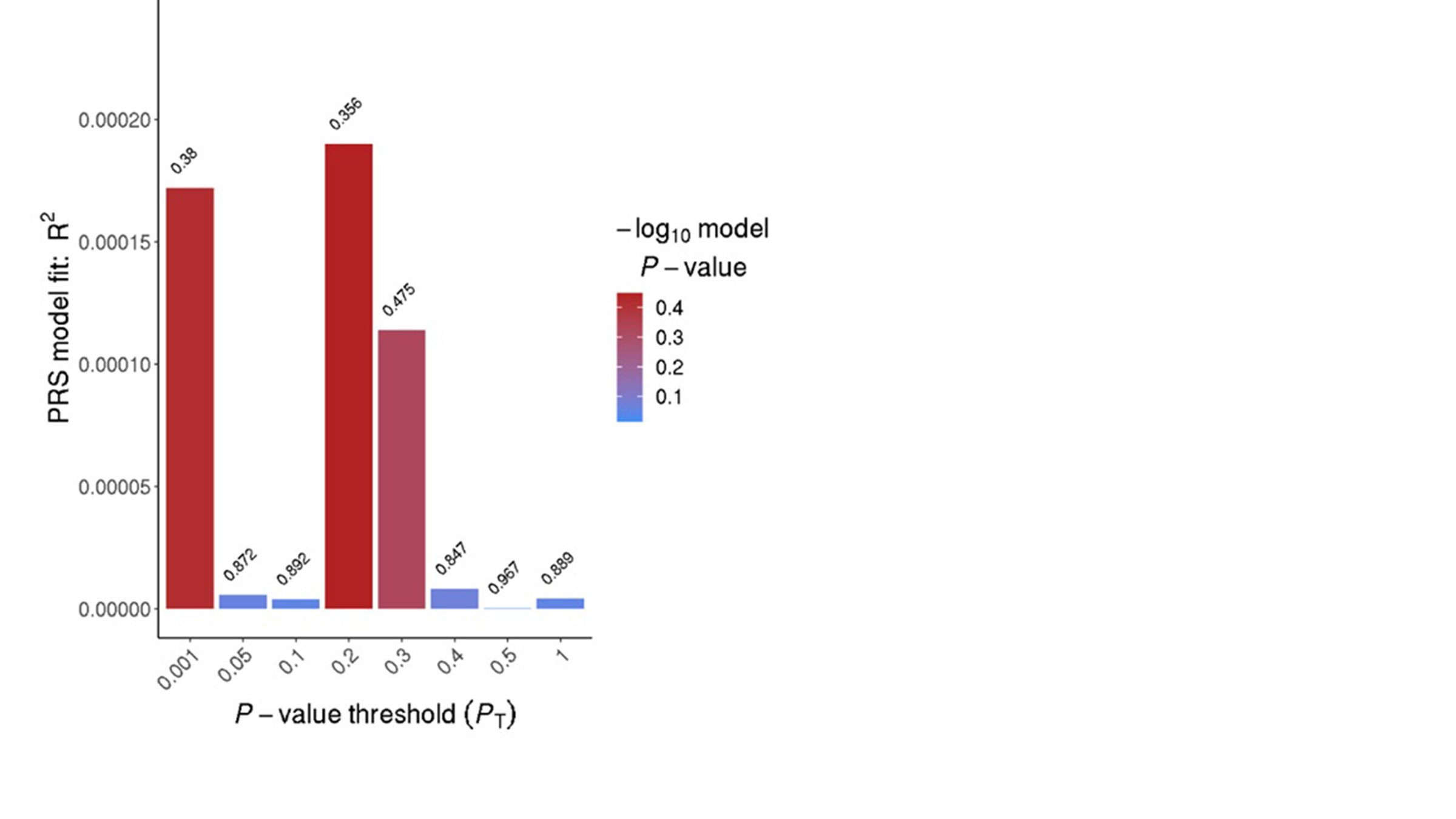

Supplement: Supplementary file 3 [file Image_2.JPEG]
