# Supplementary material for: Potential Genetic Overlap Between Insomnia and Sleep Symptoms in Major Depressive Disorder: A Polygenic Risk Score Analysis
Source: Front Psychiatry. 2021 Dec 3;12:734077. doi: 10.3389/fpsyt.2021.734077 (PMC8678563; doi:10.3389/fpsyt.2021.734077)
Supplement: Supplementary file 4 [file Data_Sheet_2.pdf]

Major Depressive Disorder Working Group of the Psychiatric Genomics Consortium

Naomi R Wray* 1, 2
Stephan Ripke* 3, 4, 5
Manuel Mattheisen* 6, 7, 8
Maciej Trzaskowski 1
Enda M Byrne 1
Abdel Abdellaoui 9
Mark J Adams 10
Esben Agerbo 11, 12, 13
Tracy M Air 14
Till F M Andlauer 15, 16
Silviu-Alin Bacanu 17
Marie Bækvad-Hansen 13, 18
Aartjan T F Beekman 19
Tim B Bigdeli 17, 20
Elisabeth B Binder 15, 21
Julien Bryois 22
Henriette N Buttenschøn 13, 23, 24
Jonas Bybjerg-Grauholm 13, 18
Na Cai 25, 26
Enrique Castelao 27
Jane Hvarregaard Christensen 8, 13, 24
Toni-Kim Clarke 10
Jonathan R I Coleman 28
Lucía Colodro-Conde 29
Baptiste Couvy-Duchesne 2, 30
Nick Craddock 31
Gregory E Crawford 32, 33
Gail Davies 34
Franziska Degenhardt 35
Eske M Derks 29
Nese Direk 36, 37
Conor V Dolan 9
Erin C Dunn 38, 39, 40
Thalia C Eley 28
Valentina Escott-Price 41
Farnush Farhadi Hassan Kiadeh 42
Hilary K Finucane 43, 44
Jerome C Foo 45
Andreas J Forstner 35, 46, 47, 48
Josef Frank 45
Héléna A Gaspar 28
Michael Gill 49
Fernando S Goes 50
Scott D Gordon 29
Jakob Grove 8, 13, 24, 51
Lynsey S Hall 10, 52
Christine Søholm Hansen 13, 18
Thomas F Hansen 53, 54, 55
Stefan Herms 35, 47
Ian B Hickie 56
Per Hoffmann 35, 47
Georg Homuth 57
Carsten Horn 58
Jouke-Jan Hottenga 9
David M Hougaard 13, 18
David M Howard 10, 28
Marcus Ising 59
Rick Jansen 19
Ian Jones 60
Lisa A Jones 61
Eric Jorgenson 62
James A Knowles 63
Isaac S Kohane 64, 65, 66
Julia Kraft 4
Warren W. Kretzschmar 67
Zoltán Kutalik 68, 69
Yihan Li 67
Penelope A Lind 29
Donald J MacIntyre 70, 71
Dean F MacKinnon 50
Robert M Maier 2
Wolfgang Maier 72
Jonathan Marchini 73
Hamdi Mbarek 9
Patrick McGrath 74
Peter McGuffin 28
Sarah E Medland 29
Divya Mehta 2, 75
Christel M Middeldorp 9, 76, 77
Evelin Mihailov 78
Yuri Milaneschi 19
Lili Milani 78
Francis M Mondimore 50
Grant W Montgomery 1
Sara Mostafavi 79, 80
Niamh Mullins 28
Matthias Nauck 81, 82
Bernard Ng 80
Michel G Nivard 9
Dale R Nyholt 83
Paul F O'Reilly 28
Hogni Oskarsson 84
Michael J Owen 60
Jodie N Painter 29
Carsten Bøcker Pedersen 11, 12, 13
Marianne Giørtz Pedersen 11, 12, 13
Roseann E Peterson 17, 85
Wouter J Peyrot 19
Giorgio Pistis 27
Danielle Posthuma 86, 87
Jorge A Quiroz 88
Per Qvist 8, 13, 24
John P Rice 89
Brien P. Riley 17
Margarita Rivera 28, 90
Saira Saeed Mirza 36
Robert Schoevers 91
Eva C Schulte 92, 93
Ling Shen 62
Jianxin Shi 94
Stanley I Shyn 95
Engilbert Sigurdsson 96
Grant C B Sinnamon 97
Johannes H Smit 19
Daniel J Smith 98
Hreinn Stefansson 99
Stacy Steinberg 99
Fabian Streit 45
Jana Strohmaier 45
Katherine E Tansey 100
Henning Teismann 101
Alexander Teumer 102
Wesley Thompson 13, 54, 103, 104
Pippa A Thomson 105
Thorgeir E Thorgeirsson 99
Matthew Traylor 106
Jens Treutlein 45
Vassily Trubetskoy 4
André G Uitterlinden 107
Daniel Umbricht 108
Sandra Van der Auwera 109
Albert M van Hemert 110
Alexander Viktorin 22
Peter M Visscher 1, 2
Yunpeng Wang 13, 54, 104
Bradley T. Webb 111
Shantel Marie Weinsheimer 13, 54
Jürgen Wellmann 101
Gonneke Willemsen 9

Stephanie H Witt 45
Yang Wu 1
Hualin S Xi 112
Jian Yang 2, 113
Futao Zhang 1
Volker Arolt 114
Bernhard T Baune 114, 115, 116
Klaus Berger 101
Dorret I Boomsma 9
Sven Cichon 35, 47, 117, 118
Udo Dannlowski 114
EJC de Geus 9, 119
J Raymond DePaulo 50
Enrico Domenici 120
Katharina Domschke 121, 122
Tõnu Esko 5, 78
Hans J Grabe 109
Steven P Hamilton 123
Caroline Hayward 124
Andrew C Heath 89
Kenneth S Kendler 17
Stefan Kloiber 59, 125, 126
Glyn Lewis 127
Qingqin S Li 128
Susanne Lucae 59
Pamela AF Madden 89
Patrik K Magnusson 22
Nicholas G Martin 29
Andrew M McIntosh 10, 34
Andres Metspalu 78, 129
Ole Mors 13, 130
Preben Bo Mortensen 11, 12, 13, 24
Bertram Müller-Myhsok 15, 131, 132
Merete Nordentoft 13, 133
Markus M Nöthen 35
Michael C O'Donovan 60
Sara A Paciga 134
Nancy L Pedersen 22
Brenda WJH Penninx 19
Roy H Perlis 38, 135
David J Porteous 105
James B Potash 136
Martin Preisig 27
Marcella Rietschel 45
Catherine Schaefer 62
Thomas G Schulze 45, 93, 137, 138, 139
Jordan W Smoller 38, 39, 40
Kari Stefansson 99, 140
Henning Tiemeier 36, 141, 142
Rudolf Uher 143
Henry Völzke 102
Myrna M Weissman 74, 144
Thomas Werge 13, 54, 145
Cathryn M Lewis* 28, 146
Douglas F Levinson* 147
Gerome Breen* 28, 148
Anders D Børglum* 8, 13, 24
Patrick F Sullivan* 22, 149, 150

1, Institute for Molecular Bioscience, The University of Queensland, Brisbane, QLD, AU
2, Queensland Brain Institute, The University of Queensland, Brisbane, QLD, AU
3, Analytic and Translational Genetics Unit, Massachusetts General Hospital, Boston, MA, US
4, Department of Psychiatry and Psychotherapy, Universitätsmedizin Berlin Campus Charité Mitte, Berlin, DE
5, Medical and Population Genetics, Broad Institute, Cambridge, MA, US
6, Department of Psychiatry, Psychosomatics and Psychotherapy, University of Wurzburg, Wurzburg, DE
7, Centre for Psychiatry Research, Department of Clinical Neuroscience, Karolinska Institutet, Stockholm, SE
8, Department of Biomedicine, Aarhus University, Aarhus, DK
9, Dept of Biological Psychology & EMGO+ Institute for Health and Care Research, Vrije Universiteit Amsterdam, Amsterdam, NL
10, Division of Psychiatry, University of Edinburgh, Edinburgh, GB
11, Centre for Integrated Register-based Research, Aarhus University, Aarhus, DK
12, National Centre for Register-Based Research, Aarhus University, Aarhus, DK
13, iPSYCH, The Lundbeck Foundation Initiative for Integrative Psychiatric Research,, DK
14, Discipline of Psychiatry, University of Adelaide, Adelaide, SA, AU
15, Department of Translational Research in Psychiatry, Max Planck Institute of Psychiatry, Munich, DE
16, Department of Neurology, Klinikum rechts der Isar, Technical University of Munich, Munich, DE
17, Department of Psychiatry, Virginia Commonwealth University, Richmond, VA, US
18, Center for Neonatal Screening, Department for Congenital Disorders, Statens Serum Institut, Copenhagen, DK
19, Department of Psychiatry, Vrije Universiteit Medical Center and GGZ inGeest, Amsterdam, NL
20, Virginia Institute for Psychiatric and Behavior Genetics, Richmond, VA, US
21, Department of Psychiatry and Behavioral Sciences, Emory University School of Medicine, Atlanta, GA, US
22, Department of Medical Epidemiology and Biostatistics, Karolinska Institutet, Stockholm, SE
23, Department of Clinical Medicine, Translational Neuropsychiatry Unit, Aarhus University, Aarhus, DK
24, iSEQ, Centre for Integrative Sequencing, Aarhus University, Aarhus, DK
25, Human Genetics, Wellcome Trust Sanger Institute, Cambridge, GB
26, Statistical genomics and systems genetics, European Bioinformatics Institute (EMBL-EBI), Cambridge, GB
27, Department of Psychiatry, Lausanne University Hospital and University of Lausanne, Lausanne, CH
28, Social, Genetic and Developmental Psychiatry Centre, King's College London, London, GB
29, Genetics and Computational Biology, QIMR Berghofer Medical Research Institute, Brisbane, QLD, AU
30, Centre for Advanced Imaging, The University of Queensland, Brisbane, QLD, AU
31, Psychological Medicine, Cardiff University, Cardiff, GB
32, Center for Genomic and Computational Biology, Duke University, Durham, NC, US
33, Department of Pediatrics, Division of Medical Genetics, Duke University, Durham, NC, US
34, Centre for Cognitive Ageing and Cognitive Epidemiology, University of Edinburgh, Edinburgh, GB
35, Institute of Human Genetics, University of Bonn, School of Medicine & University Hospital Bonn, Bonn, DE
36, Epidemiology, Erasmus MC, Rotterdam, Zuid-Holland, NL
37, Psychiatry, Dokuz Eylul University School Of Medicine, Izmir, TR
38, Department of Psychiatry, Massachusetts General Hospital, Boston, MA, US
39, Psychiatric and Neurodevelopmental Genetics Unit (PNGU), Massachusetts General Hospital, Boston, MA, US
40, Stanley Center for Psychiatric Research, Broad Institute, Cambridge, MA, US
41, Neuroscience and Mental Health, Cardiff University, Cardiff, GB
42, Bioinformatics, University of British Columbia, Vancouver, BC, CA
43, Department of Epidemiology, Harvard T.H. Chan School of Public Health, Boston, MA, US
44, Department of Mathematics, Massachusetts Institute of Technology, Cambridge, MA, US
45, Department of Genetic Epidemiology in Psychiatry, Central Institute of Mental Health, Medical Faculty Mannheim, Heidelberg University, Mannheim, Baden-Württemberg, DE
46, Department of Psychiatry (UPK), University of Basel, Basel, CH
47, Department of Biomedicine, University of Basel, Basel, CH
48, Centre for Human Genetics, University of Marburg, Marburg, DE

49, Department of Psychiatry, Trinity College Dublin, Dublin, IE
50, Psychiatry & Behavioral Sciences, Johns Hopkins University, Baltimore, MD, US
51, Bioinformatics Research Centre, Aarhus University, Aarhus, DK
52, Institute of Genetic Medicine, Newcastle University, Newcastle upon Tyne, GB
53, Danish Headache Centre, Department of Neurology, Rigshospitalet, Glostrup, DK
54, Institute of Biological Psychiatry, Mental Health Center Sct. Hans, Mental Health Services Capital Region of Denmark, Copenhagen, DK
55, iPSYCH, The Lundbeck Foundation Initiative for Psychiatric Research, Copenhagen, DK
56, Brain and Mind Centre, University of Sydney, Sydney, NSW, AU
57, Interfaculty Institute for Genetics and Functional Genomics, Department of Functional Genomics, University Medicine and Ernst Moritz Arndt University Greifswald, Greifswald, Mecklenburg-Vorpommern, DE
58, Roche Pharmaceutical Research and Early Development, Pharmaceutical Sciences, Roche Innovation Center Basel, F. Hoffmann-La Roche Ltd, Basel, CH
59, Max Planck Institute of Psychiatry, Munich, DE
60, MRC Centre for Neuropsychiatric Genetics and Genomics, Cardiff University, Cardiff, GB
61, Department of Psychological Medicine, University of Worcester, Worcester, GB
62, Division of Research, Kaiser Permanente Northern California, Oakland, CA, US
63, Psychiatry & The Behavioral Sciences, University of Southern California, Los Angeles, CA, US
64, Department of Biomedical Informatics, Harvard Medical School, Boston, MA, US
65, Department of Medicine, Brigham and Women's Hospital, Boston, MA, US
66, Informatics Program, Boston Children's Hospital, Boston, MA, US
67, Wellcome Trust Centre for Human Genetics, University of Oxford, Oxford, GB
68, Institute of Social and Preventive Medicine (IUMSP), Lausanne University Hospital and University of Lausanne, Lausanne, VD, CH
69, Swiss Institute of Bioinformatics, Lausanne, VD, CH
70, Division of Psychiatry, Centre for Clinical Brain Sciences, University of Edinburgh, Edinburgh, GB
71, Mental Health, NHS 24, Glasgow, GB
72, Department of Psychiatry and Psychotherapy, University of Bonn, Bonn, DE
73, Statistics, University of Oxford, Oxford, GB
74, Psychiatry, Columbia University College of Physicians and Surgeons, New York, NY, US
75, School of Psychology and Counseling, Queensland University of Technology, Brisbane, QLD, AU
76, Child and Youth Mental Health Service, Children's Health Queensland Hospital and Health Service, South Brisbane, QLD, AU
77, Child Health Research Centre, University of Queensland, Brisbane, QLD, AU
78, Estonian Genome Center, University of Tartu, Tartu, EE
79, Medical Genetics, University of British Columbia, Vancouver, BC, CA
80, Statistics, University of British Columbia, Vancouver, BC, CA
81, DZHK (German Centre for Cardiovascular Research), Partner Site Greifswald, University Medicine, University Medicine Greifswald, Greifswald, Mecklenburg-Vorpommern, DE
82, Institute of Clinical Chemistry and Laboratory Medicine, University Medicine Greifswald, Greifswald, Mecklenburg-Vorpommern, DE
83, Institute of Health and Biomedical Innovation, Queensland University of Technology, Brisbane, QLD, AU
84, Humus, Reykjavik, IS
85, Virginia Institute for Psychiatric & Behavioral Genetics, Virginia Commonwealth University, Richmond, VA, US
86, Clinical Genetics, Vrije Universiteit Medical Center, Amsterdam, NL
87, Complex Trait Genetics, Vrije Universiteit Amsterdam, Amsterdam, NL
88, Solid Biosciences, Boston, MA, US
89, Department of Psychiatry, Washington University in Saint Louis School of Medicine, Saint Louis, MO, US
90, Department of Biochemistry and Molecular Biology II, Institute of Neurosciences, Biomedical Research Center (CIBM), University of Granada, Granada, ES

91, Department of Psychiatry, University of Groningen, University Medical Center Groningen, Groningen, NL
92, Department of Psychiatry and Psychotherapy, University Hospital, Ludwig Maximilian University Munich, Munich, DE
93, Institute of Psychiatric Phenomics and Genomics (IPPG), University Hospital, Ludwig Maximilian University Munich, Munich, DE
94, Division of Cancer Epidemiology and Genetics, National Cancer Institute, Bethesda, MD, US
95, Behavioral Health Services, Kaiser Permanente Washington, Seattle, WA, US
96, Faculty of Medicine, Department of Psychiatry, University of Iceland, Reykjavik, IS
97, School of Medicine and Dentistry, James Cook University, Townsville, QLD, AU
98, Institute of Health and Wellbeing, University of Glasgow, Glasgow, GB
99, deCODE Genetics / Amgen, Reykjavik, IS
100, College of Biomedical and Life Sciences, Cardiff University, Cardiff, GB
101, Institute of Epidemiology and Social Medicine, University of Münster, Münster, Nordrhein-Westfalen, DE
102, Institute for Community Medicine, University Medicine Greifswald, Greifswald, Mecklenburg-Vorpommern, DE
103, Department of Psychiatry, University of California, San Diego, San Diego, CA, US
104, KG Jebsen Centre for Psychosis Research, Norway Division of Mental Health and Addiction, Oslo University Hospital, Oslo, NO
105, Medical Genetics Section, CGEM, IGMM, University of Edinburgh, Edinburgh, GB
106, Clinical Neurosciences, University of Cambridge, Cambridge, GB
107, Internal Medicine, Erasmus MC, Rotterdam, Zuid-Holland, NL
108, Roche Pharmaceutical Research and Early Development, Neuroscience, Ophthalmology and Rare Diseases Discovery & Translational Medicine Area, Roche Innovation Center Basel, F. Hoffmann-La Roche Ltd, Basel, CH
109, Department of Psychiatry and Psychotherapy, University Medicine Greifswald, Greifswald, Mecklenburg-Vorpommern, DE
110, Department of Psychiatry, Leiden University Medical Center, Leiden, NL
111, Virginia Institute for Psychiatric & Behavioral Genetics, Virginia Commonwealth University, Richmond, VA, US
112, Computational Sciences Center of Emphasis, Pfizer Global Research and Development, Cambridge, MA, US
113, Institute for Molecular Bioscience; Queensland Brain Institute, The University of Queensland, Brisbane, QLD, AU
114, Department of Psychiatry, University of Münster, Münster, Nordrhein-Westfalen, DE
115, Department of Psychiatry, Melbourne Medical School, University of Melbourne, Melbourne, AU
116, Florey Institute for Neuroscience and Mental Health, University of Melbourne, Melbourne, AU
117, Institute of Medical Genetics and Pathology, University Hospital Basel, University of Basel, Basel, CH
118, Institute of Neuroscience and Medicine (INM-1), Research Center Juelich, Juelich, DE
119, Amsterdam Public Health Institute, Vrije Universiteit Medical Center, Amsterdam, NL
120, Centre for Integrative Biology, Università degli Studi di Trento, Trento, Trentino-Alto Adige, IT
121, Department of Psychiatry and Psychotherapy, Medical Center - University of Freiburg, Faculty of Medicine, University of Freiburg, Freiburg, DE
122, Center for NeuroModulation, Faculty of Medicine, University of Freiburg, Freiburg, DE
123, Psychiatry, Kaiser Permanente Northern California, San Francisco, CA, US
124, Medical Research Council Human Genetics Unit, Institute of Genetics and Molecular Medicine, University of Edinburgh, Edinburgh, GB
125, Department of Psychiatry, University of Toronto, Toronto, ON, CA
126, Centre for Addiction and Mental Health, Toronto, ON, CA
127, Division of Psychiatry, University College London, London, GB
128, Neuroscience Therapeutic Area, Janssen Research and Development, LLC, Titusville, NJ, US
129, Institute of Molecular and Cell Biology, University of Tartu, Tartu, EE
130, Psychosis Research Unit, Aarhus University Hospital, Risskov, Aarhus, DK
131, Munich Cluster for Systems Neurology (SyNergy), Munich, DE
132, University of Liverpool, Liverpool, GB

133, Mental Health Center Copenhagen, Copenhagen Universtity Hospital, Copenhagen, DK
134, Human Genetics and Computational Biomedicine, Pfizer Global Research and Development, Groton, CT, US
135, Psychiatry, Harvard Medical School, Boston, MA, US
136, Psychiatry, University of Iowa, Iowa City, IA, US
137, Department of Psychiatry and Behavioral Sciences, Johns Hopkins University, Baltimore, MD, US
138, Department of Psychiatry and Psychotherapy, University Medical Center Göttingen, Goettingen, Niedersachsen, DE
139, Human Genetics Branch, NIMH Division of Intramural Research Programs, Bethesda, MD, US
140, Faculty of Medicine, University of Iceland, Reykjavik, IS
141, Child and Adolescent Psychiatry, Erasmus MC, Rotterdam, Zuid-Holland, NL
142, Psychiatry, Erasmus MC, Rotterdam, Zuid-Holland, NL
143, Psychiatry, Dalhousie University, Halifax, NS, CA
144, Division of Translational Epidemiology, New York State Psychiatric Institute, New York, NY, US
145, Department of Clinical Medicine, University of Copenhagen, Copenhagen, DK
146, Department of Medical & Molecular Genetics, King's College London, London, GB
147, Psychiatry & Behavioral Sciences, Stanford University, Stanford, CA, US
148, NIHR Maudsley Biomedical Research Centre, King's College London, London, GB
149, Genetics, University of North Carolina at Chapel Hill, Chapel Hill, NC, US
150, Psychiatry, University of North Carolina at Chapel Hill, Chapel Hill, NC, US
